# Transient Cerebral Ischemia Promotes Brain Mitochondrial Dysfunction and Exacerbates Cognitive Impairments in Young 5xFAD Mice

**DOI:** 10.1371/journal.pone.0144068

**Published:** 2015-12-03

**Authors:** Lin Lu, Lan Guo, Esha Gauba, Jing Tian, Lu Wang, Neha Tandon, Malini Shankar, Simon J. Beck, Yifeng Du, Heng Du

**Affiliations:** 1 Department of Biological Sciences, University of Texas at Dallas, Richardson, Texas, United States of America; 2 Department of Neurology, Shandong Provincial Hospital Affiliated to Shandong University, Jinan, Shandong, The People’s Republic of China; Nathan Kline Institute and New York University Langone Medical Center, UNITED STATES

## Abstract

Alzheimer's disease (AD) is heterogeneous and multifactorial neurological disorder; and the risk factors of AD still remain elusive. Recent studies have highlighted the role of vascular factors in promoting the progression of AD and have suggested that ischemic events increase the incidence of AD. However, the detailed mechanisms linking ischemic insult to the progression of AD is still largely undetermined. In this study, we have established a transient cerebral ischemia model on young 5xFAD mice and their non-transgenic (nonTg) littermates by the transient occlusion of bilateral common carotid arteries. We have found that transient cerebral ischemia significantly exacerbates brain mitochondrial dysfunction including mitochondrial respiration deficits, oxidative stress as well as suppressed levels of mitochondrial fusion proteins including optic atrophy 1 (OPA1) and mitofusin 2 (MFN2) in young 5xFAD mice resulting in aggravated spatial learning and memory. Intriguingly, transient cerebral ischemia did not induce elevation in the levels of cortical or mitochondrial Amyloid beta (Aβ)1-40 or 1–42 levels in 5xFAD mice. In addition, the glucose- and oxygen-deprivation-induced apoptotic neuronal death in Aβ-treated neurons was significantly mitigated by mitochondria-targeted antioxidant mitotempo which suppresses mitochondrial superoxide levels. Therefore, the simplest interpretation of our results is that young 5xFAD mice with pre-existing AD-like mitochondrial dysfunction are more susceptible to the effects of transient cerebral ischemia; and ischemic events may exacerbate dementia and worsen the outcome of AD patients by exacerbating mitochondrial dysfunction.

## Introduction

Alzheimer's disease (AD) is a chronic neurodegenerative disorder characterized by progressive cognitive decline in the patients [1, 2]. Although AD is the most common type of dementia and has been intensively studied for years, the risk factors of this neurological disorder still remain largely undetermined. In past years, a growing number of epidemiologic studies have highlighted vascular risk factors in AD. It has been established that cerebral hypo-perfusion is an early event among AD brain abnormalities [3]. Furthermore, several pathological states including hypertension [4], carotid atherosclerosis [5], Apolipoprotein E (ApoE) [6], and diabetic mellitus [7], which increase the incidence of cerebral ischemia, are closely linked to the development of AD. Indeed, the coincidence of transient cerebral ischemia and stroke episodes with AD has been extensively observed in AD patients at autopsy [8–10] or in AD animal models [11–13]. In addition, previous studies have also revealed that cerebral ischemia may exacerbate the pre-existing cognitive impairments in AD individuals [14]. Therefore, these findings highlight the pivotal role of vascular risk factors in the onset and progression of AD. However, the detailed mechanistic pathway linking cerebral ischemic event to the progression of AD still remains to be elucidated.

Cerebral circulation delivers oxygen, glucose, and other critical nutrients to brain, which has a heavy demand for energy in order to sustain neuronal activity [15]. Mitochondria are critical organelles that provide more than 90% of total ATP supply in neurons via mitochondrial oxidative phosphorylation by the utilization of glucose and oxygen [16]. Conceivably, compromised oxygen and glucose delivery to brain tissues may influence mitochondrial function resulting in mitochondrial dysfunction and eventually neuronal death. Indeed, severe mitochondrial dysfunction including decreased oxidative phosphorylation, increased reactive oxygen species (ROS) generation, retarded mitochondrial calcium handling capacity, and deregulated mitochondrial dynamics has been repeatedly reported in patients with stroke episodes to be a causative factor of neuronal death [17–21]. Notably, mitochondrial dysfunction is a featured early brain change in AD and closely associated with the expression of AD symptoms [2, 22–27]. Therefore, the similarity of mitochondrial dysfunction in the development of the two apparently different neurological disorders and the acute impacts of ischemia on mitochondrial function have raised an intriguing question whether cerebral ischemia confers susceptibility to AD neurodegeneration and cognitive impairments by exaggerating brain mitochondrial dysfunction.

To address this concept, we examined mitochondrial function, neurodegeneration, and cognitive function in young AD model mice (5xFAD mice) and their age-matched non-transgenic (nonTg) littermates following transient cerebral ischemia and reperfusion. 5xFAD mice are widely used in AD research. In these transgenic mice which carry mutations in Amyloid beta Precursor Protein (APP), brain Aβ deposition starts to develop in the cortex and hippocampus at 2–3 months of mouse age [28]. We used the mice at 3.5 months old when there is brain Aβ deposition with mild cognitive impairments to mimic the pre-clinical or early stage of AD [29–31]. In this study, we aim to determine whether transient cerebral ischemia promotes cognitive impairments in existing AD at its early stage by exacerbating mitochondrial dysfunction.

## Results

### Transient cerebral ischemia exacerbates mitochondrial respiration deficits in 5xFAD mice

**Fig 1:**
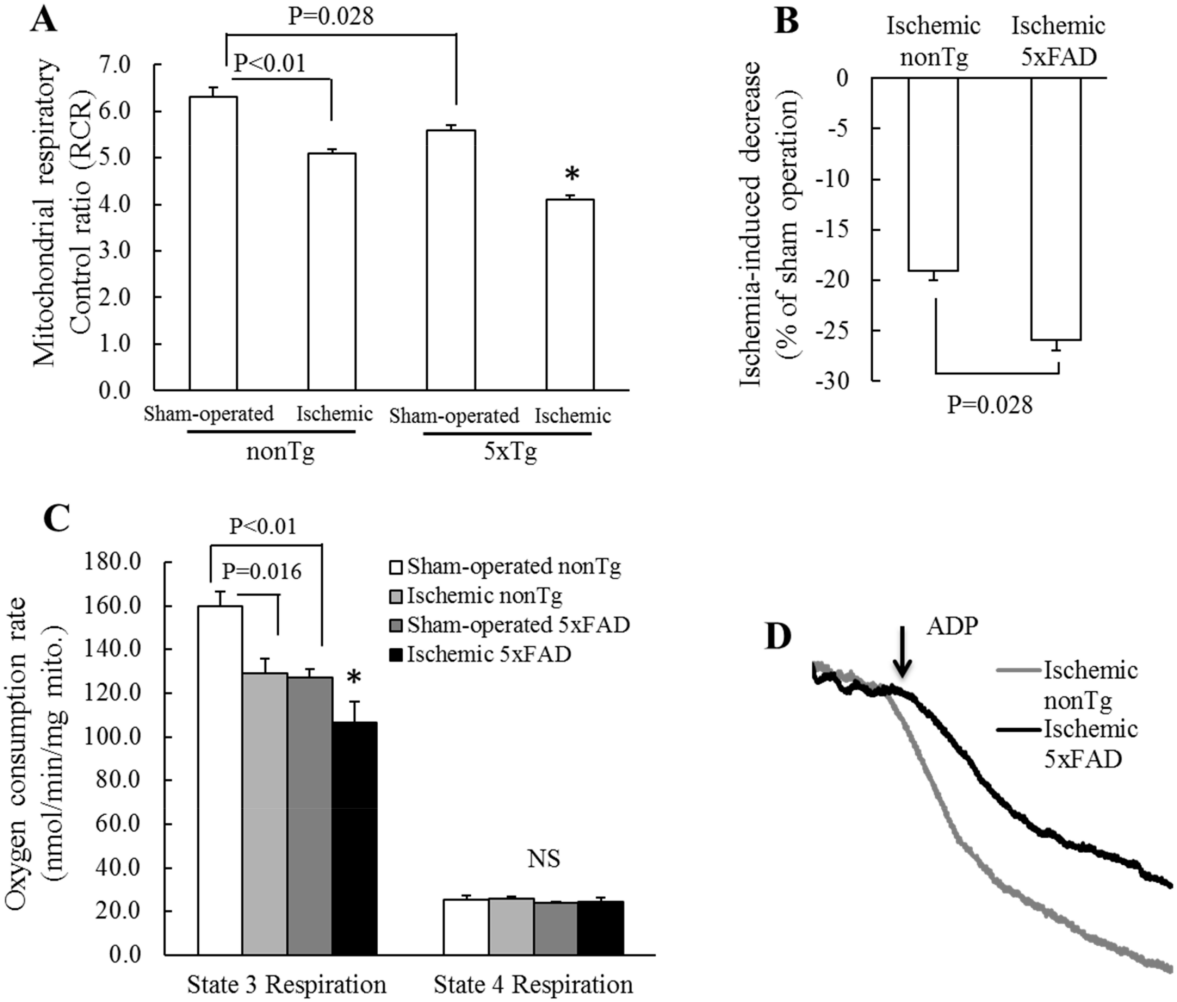
Compromised brain mitochondrial respiration in ischemic 5xFAD mice. (**A**) RCRs from the indicated groups of mice. Ischemic 5xFAD mice demonstrated substantially suppressed RCR which was significantly lowered than other groups. *P<0.05 vs other groups. (**B**) Decreased percentage of mitochondrial RCR in ischemic mice in comparison to that of their sham-operated counterpart. (**C**) Oxygen consumption rates of state 3 and state 4 respirations in the indicated groups of mice. (**D**) Representative traces of oxygen consumption of ischemic nonTg and 5xFAD mice. *P<0.05 vs other groups. N = 4–5 mice per group.

ATP provision through oxidative phosphorylation is the most important mitochondrial function [32, 33]. To assess the impact of transient cerebral ischemia on mitochondrial oxidative phosphorylation in 5xFAD mice, brain mitochondria were purified from the age-matched nonTg and 5xFAD mice at 2 weeks after transient cerebral ischemia or sham operation and subjected to the mitochondrial respiration analysis using a Clark electrode. We chose to perform experiments on mice at 2 weeks after the operation is because previous studies have shown that transient ischemia induces long-term vascular and neuronal dysfunctions which become prominent at 2 weeks after ischemic event [34–36]. Mitochondria were energized with glutamate/malate and oxygen consumption was triggered by the addition of ADP. The oxygen consumption rates in state 3 and state 4 respirations were collected and mitochondrial respiration was estimated by calculating the mitochondrial respiratory control ratio (RCR; the ratio of State 3 to State 4 respiration). Decreased mitochondrial RCR is indicative of damage in mitochondrial oxidative phosphorylation [24]. Regardless of mouse genotype, transient cerebral ischemia caused significant reduction of mitochondrial RCR in ischemic mice as compared with their sham-operated counterpart (Fig 1A) suggesting the deleterious impact of ischemic events on brain mitochondrial respiration. In addition, in comparison to sham-operated nonTg mice, the sham-operated 5xFAD mice also exhibited decreased mitochondrial RCR (Fig 1A. **P = 0.028**) demonstrating the effect of 5xFAD phenotype. When compared to their own sham-operated counterpart, ischemic 5xFAD mice exhibited a 25.96 ± 1.85% decrease in mitochondrial RCR which is significantly higher than the 19.05 ± 1.30% reduction in mitochondrial RCR between ischemic and sham-operated nonTg mice (Fig 1B. **P = 0.028**). Therefore, the result implicate that the interaction of transient cerebral ischemia and 5xFAD phenotype substantially exaggerates mitochondrial respiration deficits. It should be noted that brain mitochondria from the ischemic 5xFAD mice manifested decreased state 3 respiration with unaltered state 4 respiration in comparison to other groups of mice (Fig 1C & 1D) suggesting that their mitochondria were well preserved during purification and their reduced RCR is specifically due to dampened mitochondrial oxidative phosphorylation.

### Transient cerebral ischemia exacerbates brain oxidative stress in ischemic 5xFAD mice

**Fig 2:**
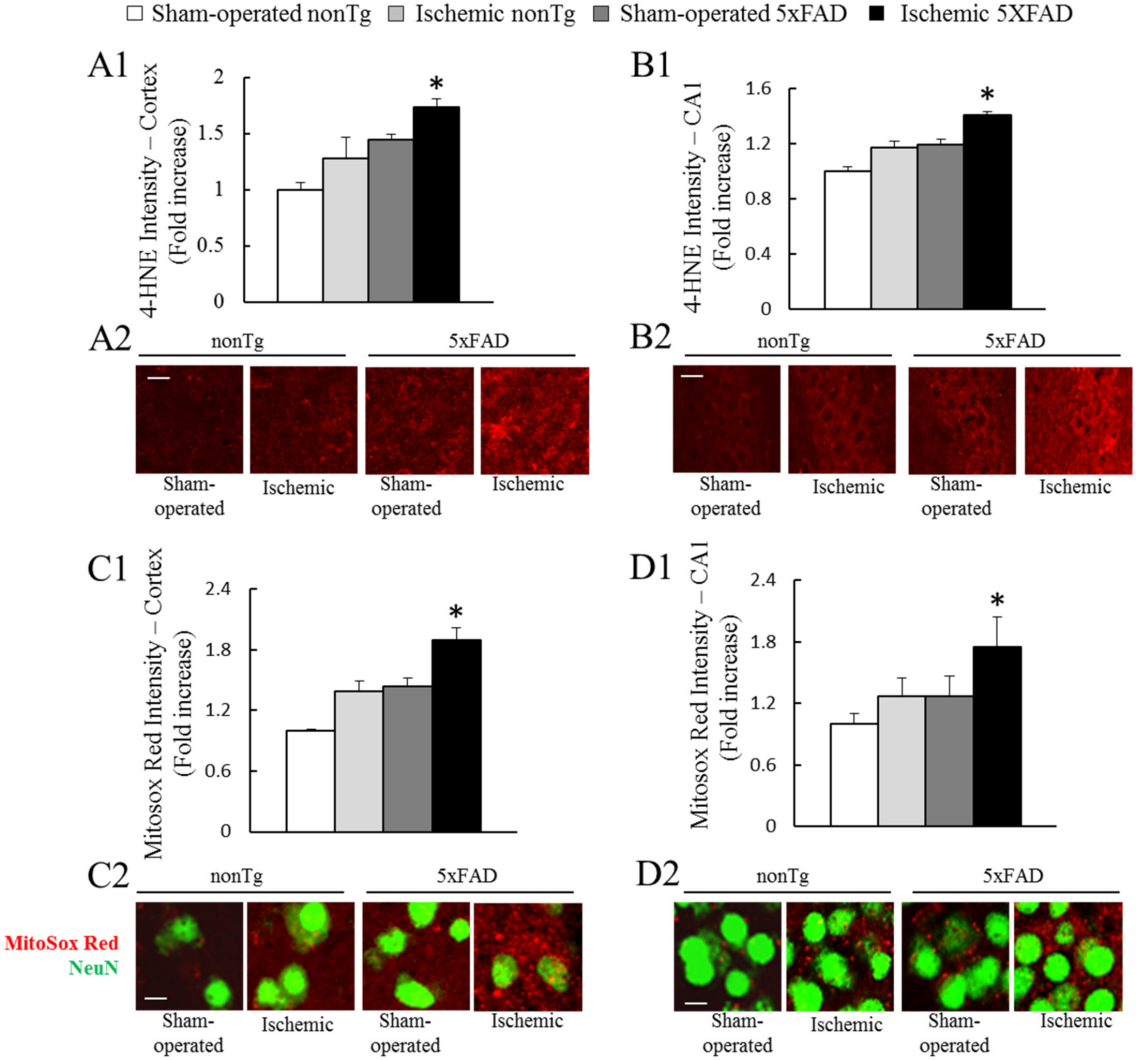
Elevated brain oxidative damage in ischemic 5xFAD mice. Immunofluorescent staining of 4-HNE in brain cortex (**A1**) and hippocampus CA1 region (**B1**) of the indicated groups of mice. *P<0.05 vs other groups. Scale bar = 20μm. (**A2**) and (**B2**) are representative images of 4-HNE staining (Red) in brain cortex and CA1 region, respectively. In situ MitoSox Red staining in brain cortex (**C1**) and hippocampus CA1 region (**D1**) of the the indicated groups of mice. *P<0.05 vs other groups. Scale bar = 10μm. (**C2**) and (**D2**) are representative images in brain cortex and CA1 region, respectively. N = 3–4 mice of each group.

In view of the deleterious impact of transient cerebral ischemia on mitochondrial oxidative phosphorylation and defected mitochondrial oxidative phosphorylation results in increased free radicals production [37], we determined whether transient cerebral ischemia worsens brain oxidative stress in 5xFAD mice. To determine oxidative stress in brains, we performed the staining of 4-hydroxy-2-nonenal (4-HNE) which is a widely accepted indicator of oxidative stress [38] on brain slices from the mice. We found significantly increased 4-HNE intensity in the cortex and hippocampus of ischemic mice in comparison to their non-ischemic controls (Fig 2A & 2B); and the change was more pronounced in ischemic 5xFAD (Fig 2A & 2B. *P<0.05 vs other groups).

Next, it is essential to determine whether the increased oxidative stress in the ischemic 5xFAD mice is associated with elevated mitochondrial reactive oxygen species (ROS) production which is thought to be the major source of neuronal oxidative stress [39]. To address this question, we examined *in situ* mitochondrial superoxide production by using Mitosox Red, a sensitive and specific fluorescent marker for mitochondrial superoxide [40]. Neurons were identified by NeuN staining. The quantitative analysis showed that ischemic nonTg and sham-operated 5xFAD demonstrated significantly increased MitoSox Red intensity in their cortex and hippocampus in comparison to sham-operated nonTg mice (Fig 2C & 2D); while the mitochondrial superoxide levels were substantially higher in ischemic 5xFAD mice than that in any other groups (Fig 2C & 2D). The results suggest that ischemic insult induces brain oxidative stress which correlates to increased mitochondrial ROS production.

### Transient cerebral ischemia impairs mitochondrial fusion proteins in ischemic 5xFAD mice

**Fig 3:**
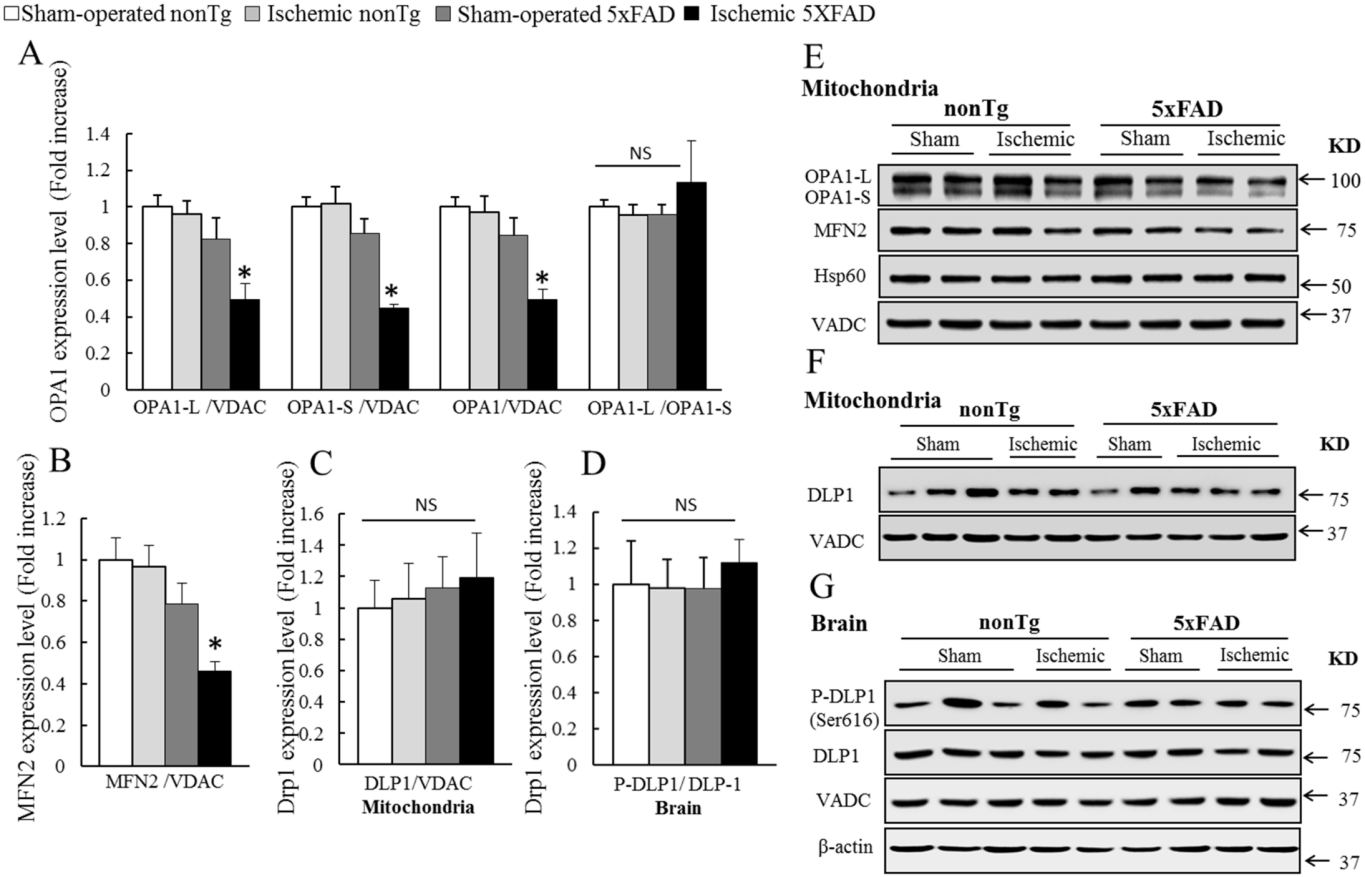
Exacerbated reduction of OPA1 and MFN2 in ischemic 5xFAD mice. (**A**) The expression levels of long OPA1 isoform (OPA1-L) and short OPA1 isoform (OPA1-S) were substantially reduced in brain mitochondria from ischemic 5xFAD mice; while the ratio of OPA1-L to OPA1-S remained unchanged. *P<0.05 vs other groups. (**B**) The expression level of MFN2 was significantly reduced in brain mitochondria from ischemic 5xFAD mice. *P<0.05 vs other groups. The levels of Dlp-1 translocation to mitochondria (**C**) or the ratios of phosphor-Dlp1 to Dlp1 in brain homogenate (**D**) in the four groups of mice were not changed. *P<0.05 vs other groups. (**E**) Representative immunoreactive bands of OPA1-L, OPA1-S and MFN2 in mitochondrial fractions. (**F**) Representative immunoreactive bands of Dlp-1 in mitochondrial fractions. (**G**) Representative immunoreactive bands of Dlp-1 and phospho-Dlp-1 in brain homogenates. Mitochondrial matrix protein Hsp60 and outer mitochondrial membrane protein VDAC were used to show the enrichment of mitochondrial fraction as well as the equal loading amount of the samples. β-actin was used to show the equal loading amount of tissue samples. N = 4–6 mice of each group.

Imbalanced mitochondrial fusion and fission is a severe mitochondrial dysfunction and increased mitochondrial fragmentation has been observed in many neurodegenerative diseases including the Alzheimer's [22, 41–43]. To determine the impact of transient cerebral ischemia on mitochondrial fusion and fission proteins in the transgenic mice *in vivo*, we first measured the expression levels of major mitochondrial fusion proteins including optic atrophy 1 (OPA1) and mitofusin 2 (MFN2) in the mitochondria isolated from the four groups of mice by immunoblotting. Brain mitochondria from ischemic 5xFAD mice exhibited marked reduction in the levels of both long and short OPA1 isoforms (Fig 3A & 3E. *P<0.05 vs other groups); while there was no significant difference in the ratio of OPA1-L/OPA1-S between any groups (Fig 3A & 3E). In addition, the level of MFN2 was substantially decreased in ischemic 5xFAD mice as well (Fig 3B & 3E. *P<0.05 vs other groups). Next, we examined the translocation of the major mitochondrial fission protein dynamin-like protein 1 (Dlp-1) to mitochondria. Interestingly, the levels of mitochondrial Dlp-1 remained unchanged in the mice at the tested age in regardless of ischemic insult or 5xFAD genotype (Fig 3C & 3F). In view of the critical role of Dlp-1 phosphorylation at Ser616 in promoting Dlp-1 translocation to mitochondria and triggering mitochondrial fission [44], we then examined the levels of phospho-Dlp-1. In agreement with the unaltered mitochondrial Dlp-1 translocation cross different groups of mice, the ratio of phospho-Dlp1 to Dlp1 was not changed in brain tissues (Fig 3D & 3G). Therefore, the results suggest that ischemic insults may break the balance of mitochondrial fusion and fission proteins which potentially affects mitochondrial dynamics and quality control.

### Lack of change in cortical and mitochondrial Aβ levels in 5xFAD mice after transient cerebral ischemia

**Fig 4:**
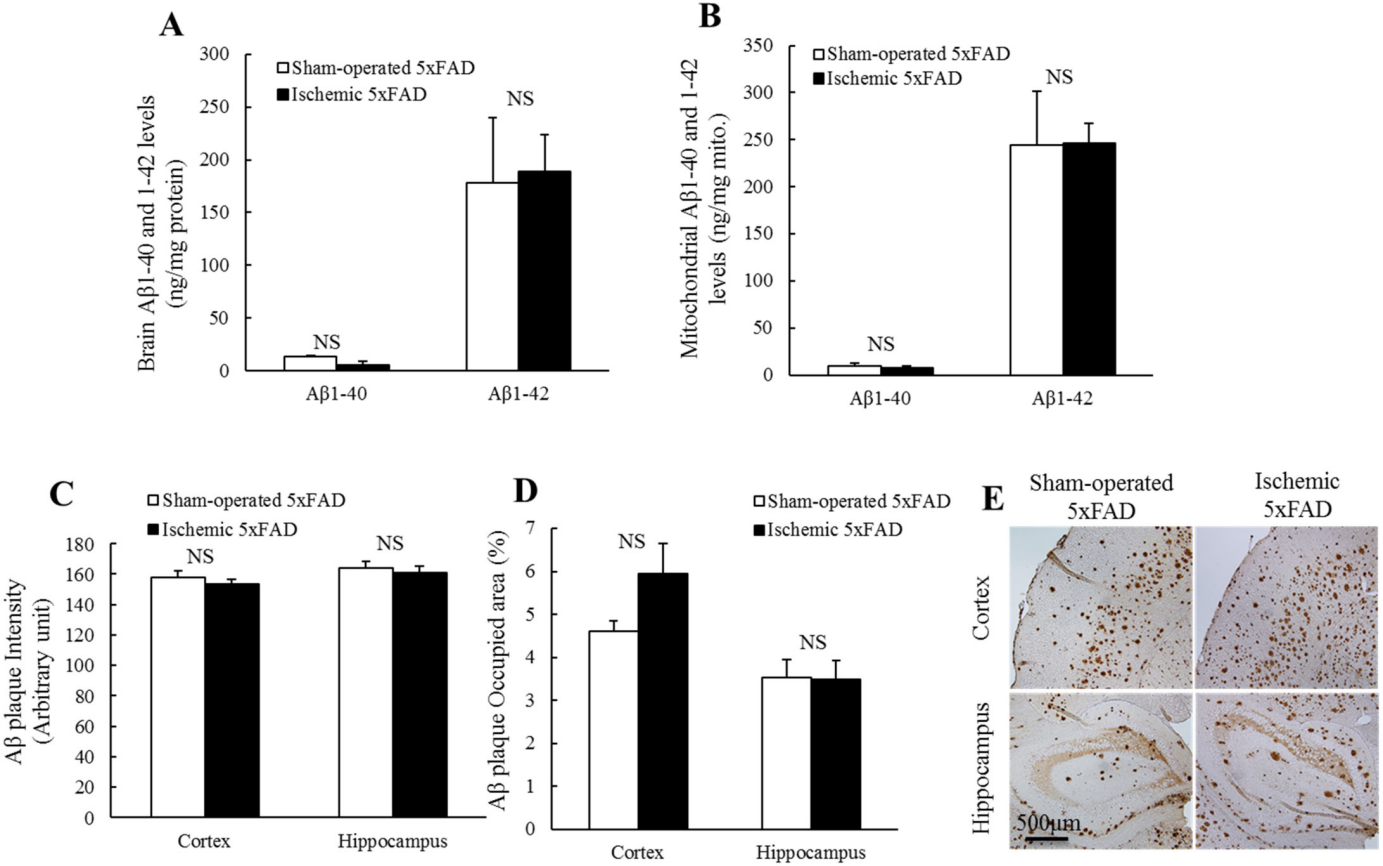
Brain and mitochondrial Aβ levels were not changed in ischemic 5xFAD mice. The levels of brain (**A**) and mitochondrial (**B**) Aβ1–40 and Aβ1–42 remained unchanged in sham-operated and ischemic 5xFAD mice. The intensity (**C**) and occupied area (%) (**D**) of Aβ plaques in cortex or hippocampus in sham-operated and ischemic 5xFAD mice were not altered. (E) Representative images of Aβ staining. Scale bar = 500 μm. N = 3–4 mice of each group.

Aβ is thought to be a key mediator of mitochondrial dysfunction in AD which is based on tons of evidence that Aβ toxicity imposes deleterious impacts on mitochondrial function [22, 23, 26, 45]. To determine whether transient cerebral ischemia affects Aβ production, we measured the levels of human Aβ 1–40 and Aβ 1–42 in the cortical extracts from sham-operated and ischemic 5xFAD mice by using ELISA assay. Quantitative analysis showed that there was no significant difference in the level of brain Aβ1–40 or Aβ1–42 between the sham-operated and ischemic 5xFAD mice (Fig 4A). Furthermore, previous reports indicate that Aβ deposits in brain mitochondria from AD cases and AD transgenic mouse models and imposes deleterious impacts on mitochondrial function [24, 46]. We then examined the levels of mitochondrial Aβ. Similar to the result of brain Aβ levels (Fig 4A), ischemic insults did not induce significant changes in the levels of mitochondrial Aβ1–40 or Aβ1–42 in 5xFAD mice (Fig 4B). Lastly, we performed experiments to compare Aβ plaques by immunohistochemistry staining using a specific antibody recognizing both Aβ1–40 and Aβ1–42. Again, our results showed that there was no significant difference in the intensity or occupied brain area of Aβ plaques in either cortex or hippocampus from the two groups of mice (Fig 4C, 4D & 4E). In together, the results suggest that transient ischemia is unlikely to alter the total brain Aβ deposition or the levels of the soluble Aβ in 5xFAD mice at least in the experimental conditions which is in agreement with previous findings [36, 47, 48].

### Transient ischemia exacerbates apoptotic cell death in ischemic 5xFAD mice

**Fig 5:**
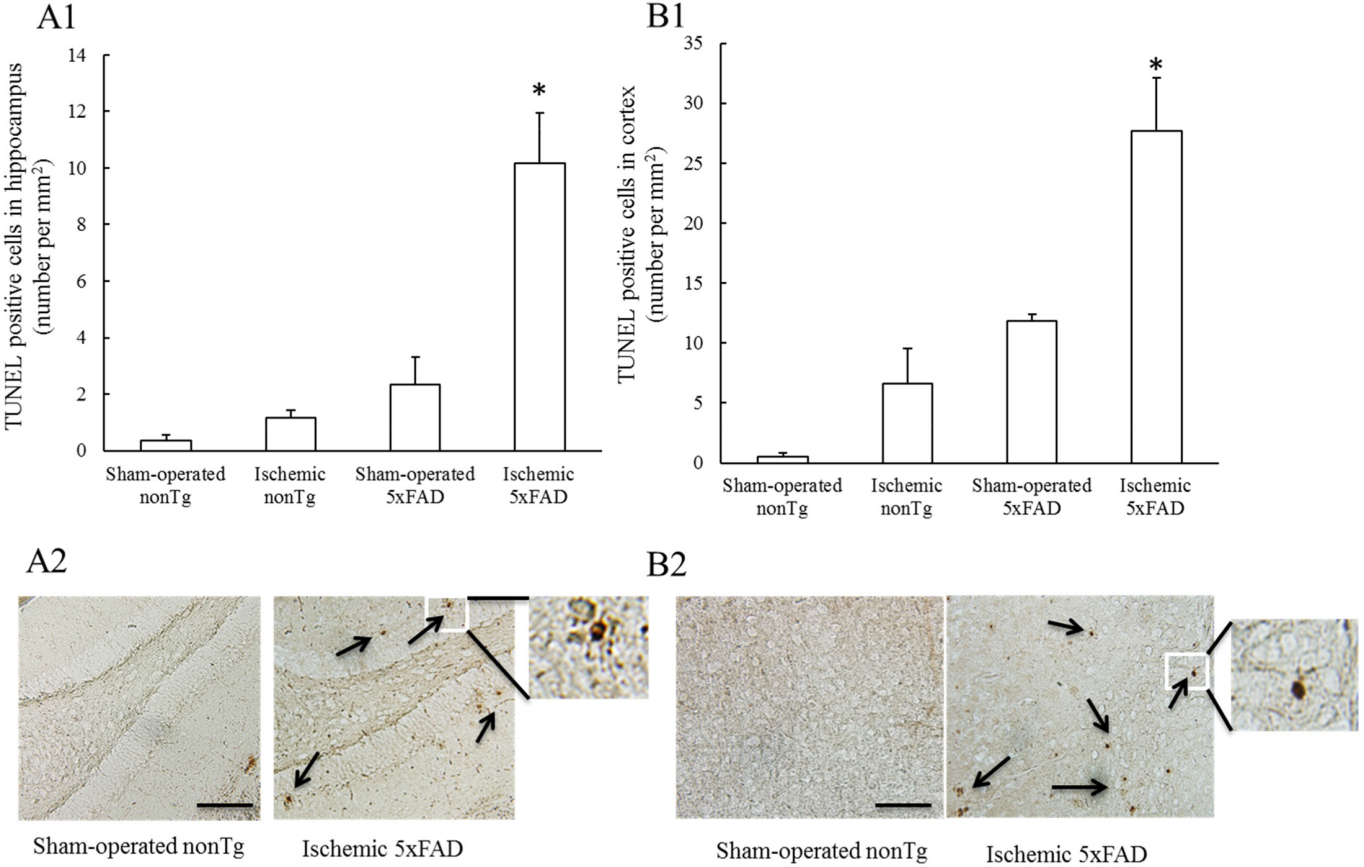
Increased apoptotic cell death in the cortex and hippocampus of ischemic 5xFAD mice. Ischemic 5xFAD mice demonstrated marked increase in the numbers of TUNEL positive cells in the cortex (**A1**) and hippocampus (**B1**). (**A2**) and (**B2**) are representative images of TUNEL staining in the cortex or hippocampus of the indicated groups of mice, respectively. Scale bar = 200 μm. Arrows indicate TUNEL positive cells. N = 3–4 mice of each group.

Since mitochondria play a critical role in supporting neuronal function and survival. Dampened mitochondrial function may lead to cell death [49], we examined the impact of ischemic insult on brain apoptotic cell death by using Terminal deoxynucleotidyl transferase dUTP nick end labeling (**TUNEL**) assay. Both ischemic nonTg and sham-operated 5xFAD mice exhibited significant increased numbers of TUNEL positive cells in the hippocampus and cortex in comparison to sham-operated nonTg mice (Fig 5A & 5B). Notably, ischemic 5xFAD mice exhibited significantly increased apoptotic cell death in hippocampus as well as brain cortex than any other groups (Fig 5A & 5B. *P<0.05).

### Transient ischemia exacerbated mouse spatial learning and memory in ischemic 5xFAD mice

**Fig 6:**
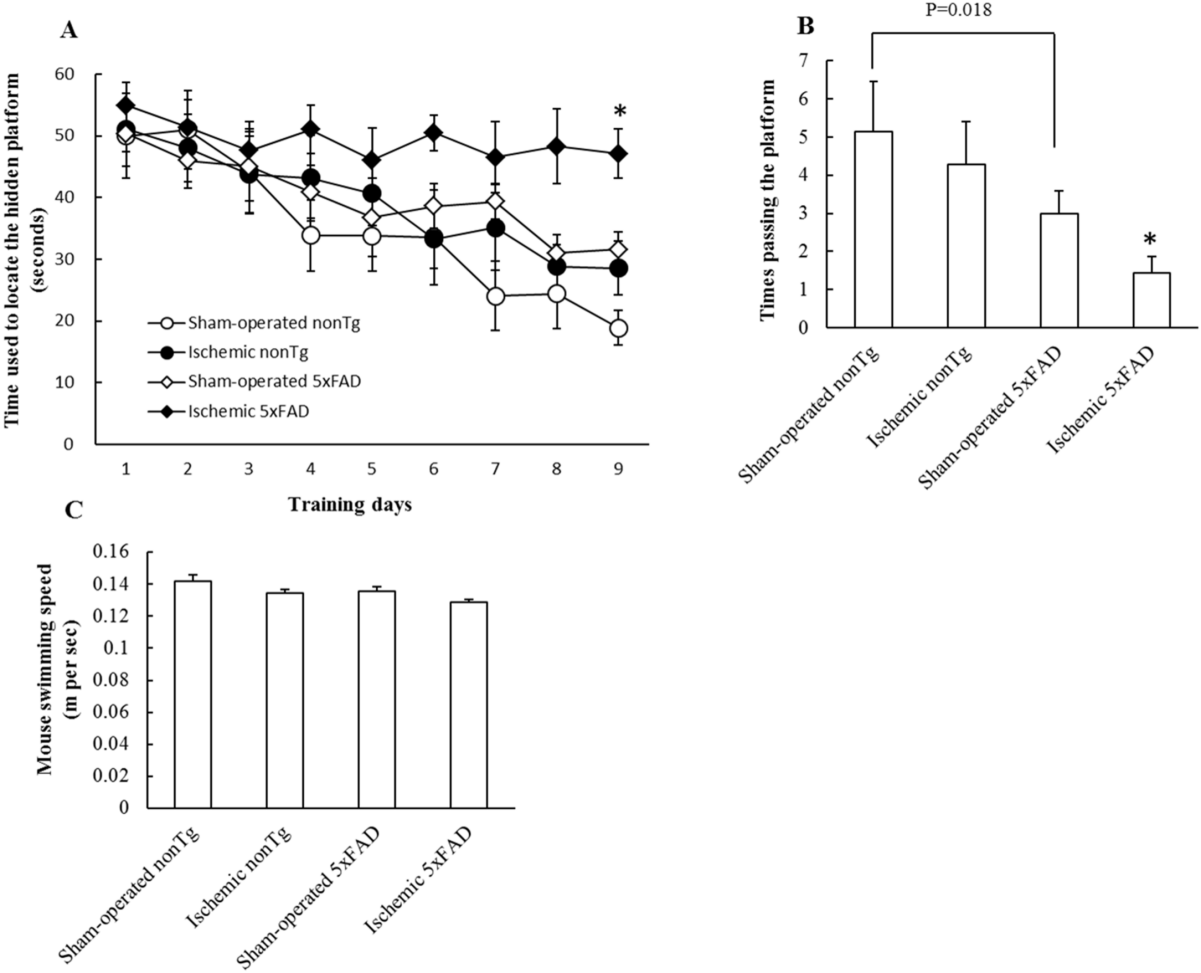
Impaired spatial learning and memory of ischemic 5xFAD mice. Ischemic 5xFAD mice demonstrated impaired learning ability to locate the hidden platform (**A**). *P<0.05 vs other groups. In addition, ischemic 5xFAD mice had compromised function in spatial reference memory (**B**). *P<0.05 vs other groups. Mice in different groups didn't show significant change in their swimming speed (**C**). N = 5–7 mice of each group.

Cognitive impairment is the outer manifestation of neuronal stress and spatial learning and memory decline is an AD-sensitive cognitive change [24]. To determine the influence of transient cerebral ischemia on 5xFAD mice spatial learning and memory, we assessed mouse spatial reference memory using a Morris Water Maze. Ischemic 5xFAD mice displayed significantly impaired spatial learning ability in sharp contrast with other groups of mice (Fig 6A. *P<0.05 vs other groups). Indeed, both ischemic nonTg mice and sham-operated 5xFAD mice showed some difficulty in finding the platform, though less severe as the ischemic 5xFAD mice, by showing increased latency time in comparison to sham-operated nonTg mice (Fig 6A). In the probe test, we removed the platform and measured the times of mouse passing the area where the hidden platform was. Ischemic nonTg mice and sham-operated 5xFAD mice passed 3–4 times within 60 seconds recording time which was less than that of sham-operated nonTg mice (~5 times); while ischemic 5xFAD mice only passed 1.4 ± 0.5 times which was substantially lessened than those of other groups (Fig 6B. *P<0.05 vs other groups). Notably, the four groups of mice did not show any significant difference in their speed of swimming (Fig 6C).

### Mitochondria-targeted antioxidant Mitotempo mitigates OGD-induced mitochondrial superoxide production increase and neuronal death

**Fig 7:**
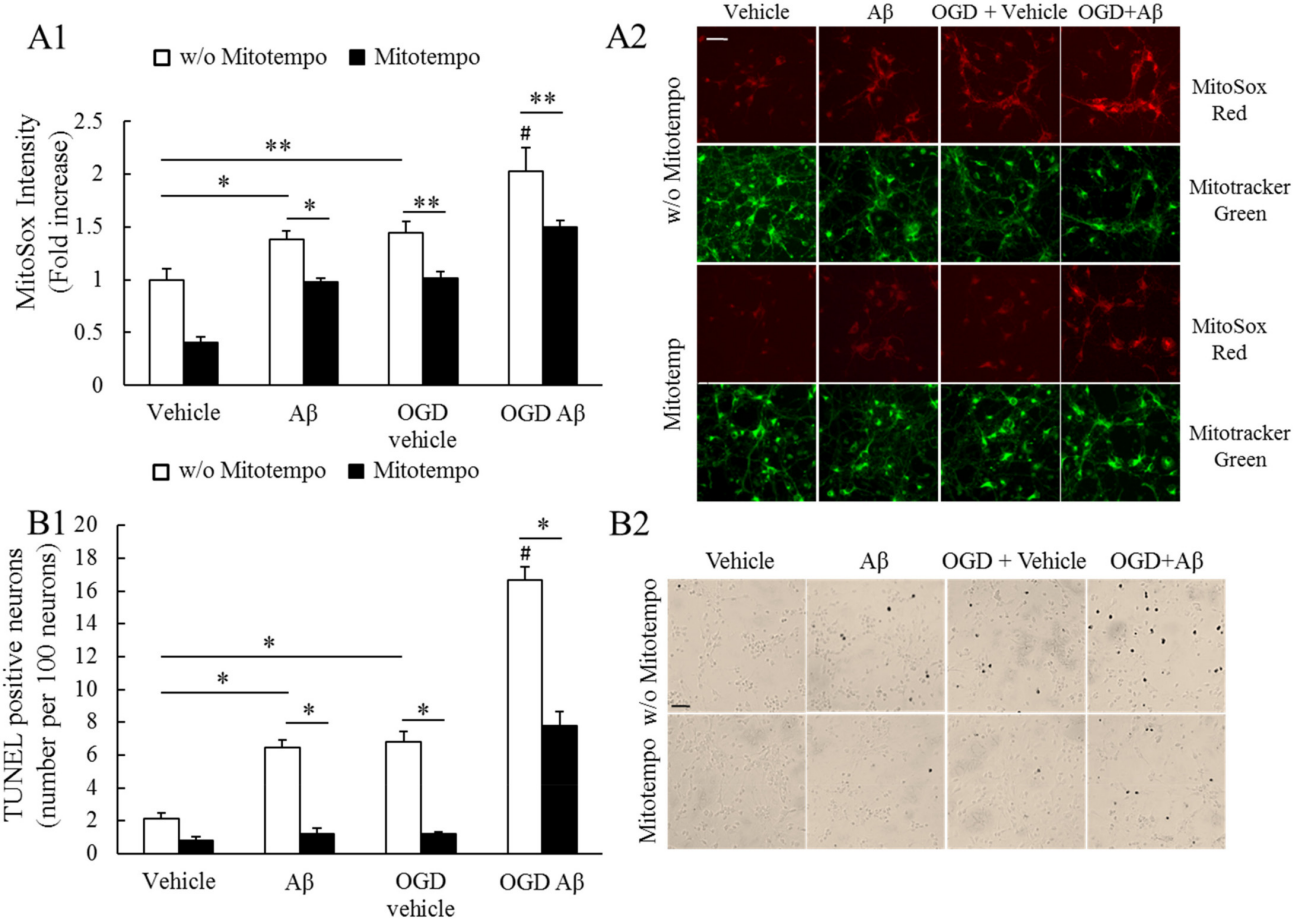
Mitochondria-targeted antioxidant Mitotempo mitigates Aβ-induced mitochondrial superoxide production and apoptotic neuronal death. (**A1**) Aβ, OGD or their combination induced significantly increased mitochondrial superoxide production which was marked attenuated by the application of Mitotempo. (**A2**) Representative images of MitoSox Red (Red) and mitotracker green (Green). Scale bar = 50μm. (**B1**) Aβ, OGD or their combination induced significantly increased apoptotic neuronal death which was marked attenuated by the application of Mitotempo. (**B2**) Representative images of TUNEL staining. Scale bar = 100μm. *P<0.05 and **P<0.01. # P<0.01 vs all the other groups.

To establish a link between transient ischemia-induced mitochondrial dysfunction as well as the resultant oxidative stress and neuronal death in Aβ-rich environments, we examined whether the suppression of mitochondrial ROS production may display protective effects. Cultured cortical neurons from nonTg pups were exposed to the treatment of Aβ at 200 nM for 24 hours before oxygen- and gluocose-deprivation (OGD). Mitochondrial superoxide level was determined by the staining of MitoSox Red [24] and mitochondria were identified by the staining of mitotracker green. Our results showed that OGD significantly elevated mitochondrial superoxide levels in neurons regardless of Aβ-treatment (Fig 7A1 & 7A2); while the changes were more severe in Aβ-treated neurons (Fig 7A1 & 7A2). In a sharp contrast, the application of a mitochondria-targeted antioxidant Mitotempo [50] substantially attenuated the OGD-induced mitochondrial ROS production (Fig 7A1 & 7A2). Next, we subjected the control or OGD-treated neurons to assess apoptotic cell death by using TUNEL assay. Similar to mitochondrial ROS detection, OGD induced significantly increased cell death which was more pronounced in Aβ-treated neurons (Fig 7B1 & 7B2) which was substantially mitigated by Mitotempo (Fig 7B1 & 7B2).

## Discussion

To understand the risk factors of the onset and progression of AD is of paramount significance for the development of therapeutic strategy for the treatment of AD. Recent studies have accentuated the role of vascular factors in the pathogenesis of AD. Emerging evidence has suggested that ischemic stroke and transient cerebral ischemia promote the pre-existing neuropathology and cognitive impairments in both AD cases [8–10] and AD animal models [11–13]. However, the detailed cellular mechanisms of transient cerebral ischemia in triggering the progression of AD are still largely unresolved. In the present study, we established transient cerebral ischemic model by subjecting mice to transient occlusion of both common carotid arteries for 20 minutes and performed experiments using the mice at 2 weeks after the procedure. The mild cognitive impairments with mild brain Aβ deposition in 5xFAD mice at the experimental age [29–31] enable us to examine the impact of the interacting insults of ischemia and Aβ on pre-existing AD-relevant mitochondrial dysfunction as well as cognitive impairments mimicking the early stage of AD. We have found that transient cerebral ischemia induces mitochondrial dysfunction and promotes brain oxidative; while such deleterious impacts of transient cerebral ischemia are substantially more prominent in 5xFAD mice. Further studies have shown that the mitochondrial deficits correlate to worsened brain apoptotic cell death and cognitive impairments in the ischemic transgenic mice. Importantly, OGD-induced neuronal death was protected by the application of a mitochondria-targeted antioxidant. Therefore, the results suggest that transient cerebral ischemia may promote the progression of AD at its early stage by promoting mitochondrial dysfunction.

Neurons take limited ATP-producing strategies which almost exclusively rely on mitochondria to generate more than 95% out of the total ATP pool [32, 33]. Conceivably, mitochondrial dysfunction results in collapsed neural functions at pathological conditions like AD. Indeed, energy failure and mitochondrial dysfunction are prominent AD brain pathology and strongly associated with synaptic degeneration and cognitive decline in AD patients as well as in AD animal models [2, 22–27]. In this study, we found that young 5xFAD mice demonstrated mild decrease in their mitochondrial respiration while the compromised mitochondrial respiration became more prominent after transient ischemia. Indeed, ischemic insult also resulted in the reduction of mitochondrial respiration in nonTg mice which is, however, less severe in comparison to ischemic 5xFAD mice. Therefore, the results suggest that mitochondria carrying pre-existing dysfunction in an Aβ rich environment are more vulnerable to the insult of transient cerebral ischemia.

A prominent deleterious consequence of defected mitochondrial respiration is the enhanced production of reactive oxygen species (ROS). Mitochondria are the major source of free radicals in neurons and damaged mitochondrial oxidative phosphorylation results in disrupted electron flow in electron transferring chain (ETC) leading to elevated generation of mitochondrial reactive oxygen species (ROS) which eventually increases oxidative stress in tissues [27]. Indeed, mitochondrial dysfunction-associated brain oxidative damage is thought to be a major causative factor of neurodegeneration in AD [27, 46, 51]. Here, we have observed dramatically exaggerated brain oxidative stress in ischemic 5xFAD mice which is in sharp contrast to either ischemic nonTg mice or sham-operated 5xFAD mice. The result conforms to our observation of the exacerbated mitochondrial respiration as well as elevated mitochondrial superoxide levels in ischemic 5xFAD mice suggesting the potential correlation of dampened mitochondrial respiration to the extensive brain and mitochondrial oxidative damages in ischemic transgenic. In addition, Mitotempo significantly protects OGD-induced apoptosis of Aβ-treated neurons further ascertaining the involving role of mitochondrial dysfunction-elevated oxidative stress in the development of neuronal stress by the interaction of ischemic insult and Aβ toxicity.

Another interesting finding of mitochondrial dysfunction in ischemic 5xFAD mice is that cortical mitochondria from the ischemic 5xFAD mice exhibited significantly reduced expression levels of OPA1 and MFN2 which are the major mitochondrial fusion proteins and their reduction is associated with compromised mitochondrial fusion capacity [52, 53]. Mitochondria are highly dynamic organelles [54]. Their morphological change achieved by constant fusion and fission is a critical mitochondrial property to accommodate to their functional state and imbalanced mitochondrial fusion and fission lead to compromised mitochondrial function and even the death of the host cells [55, 56]. Accordingly, previous studies have suggested that compromised mitochondrial fusion and activated mitochondrial fragmentation strongly correlates to neurodegeneration in many neurological disorders including stroke [57], AD [42, 58], Huntington's disease (HD) [59, 60], Parkinson's disease (PD) [61, 62], et al suggesting that impaired mitochondrial fusion is closely associated with neurodegeneration. Notably, we did not detect significant changes in the levels of Dlp-1translocation to mitochondria or the levels of phosphorylated Dlp-1 which seems to suggest that mitochondrial fusion proteins OPA1 and MFN2 are more sensitive to the ischemic insults. Given the fact that mitochondrial dynamics is maintained by the balance of fusion and fission machineries [55, 56], our results indicate that the balance of mitochondrial fusion and fission is impaired in ischemic 5xFAD mice which potentially contributes to poor mitochondrial quality control and neuronal death.

Aβ is a well-established mediator of mitochondrial dysfunction in AD. However, in this study, we found that there is no significant difference in the levels of brain or mitochondrial Aβ1-40/Aβ1–42 or the formation of cortical or hippocampal Aβ plaques between the sham-operated and ischemic 5xFAD mice. This result is in agreement with previous studies showing that transient cerebral ischemia doesn't induce Aβ overexpression [36, 47, 48]. It should be noted that several previous studies have suggested that ischemic events may promote the production of brain Aβ [13, 63]. The discrepancy may arise from the usage of different AD animal models or different experimental conditions. A comprehensive study on this issue will be conducted in our future studies.

Taken together, in this study we have determined that transient cerebral ischemia promotes mitochondrial dysfunction and exacerbates cognitive impairments in young 5xFAD mice. It should be noted that although ischemia *per se* induces similar changes in nonTg mice, the mitochondrial deficits are substantially aggravated in AD-relevant pathological settings. The results implicate that mice mimicking the early stage of AD with pre-existing AD-like mitochondrial dysfunction are more susceptible to the effects of transient cerebral ischemia. Therefore, ischemic events may exacerbate dementia and worsen the outcome of AD patients by promoting mitochondrial dysfunction.

## Materials and Methods

### Mice

Animal studies were approved by the University of Texas at Dallas Institutional Animal Care and Use Committee (IACUC) and in accordance with the National Institutes of Health guidelines for animal care. 5xFAD mice overexpress a human form of mAPP-bearing mutations (SwFlLon) and PSEN1 mutations (M146L and L286V) linked to familial AD. 5xFAD mice (B6SJL-Tg(APPSwFlLon, PSEN1*M146L*L286V) 6799Vas/Mmjax) were obtained from Jackson Laboratory. 3.5 months old male non transgenic (nonTg) or 5xFAD mice were randomly allocated into four groups based on genotyping. The investigators performing the experiments did not select the mice allocation. The number of mice was determined by our previous data and power calculation. Statistical Analysis was performed to determine whether the combinations of different types of mice are statistically different for experimental designs. If the analysis indicated it was valid to continue, individual group and the combinations of group × treatment were compared by use of the Bonferroni corrected t-test. Based on our design, we performed power calculation to see whether we have enough power to detect the differences (http://www.stat.uiowa.edu/~rlenth/Power/).

### Transient occlusion of bilateral common carotid arteries

The procedure to induce transient cerebral ischemia was conducted as previously described [64, 65]. Briefly, surgical anesthesia was induced with isoflurane (3%) in a mixture of nitrous oxide and oxygen (70:30). Anesthesia was maintained throughout the procedure with isoflurane (1.2–1.7%) delivered via a face mask. Rectal temperature was monitored and the body temperature was maintained at 37±0.5°C by using a heating pad. An anterior midline incision was made in the neck, and both common carotid arteries were then exposed and loosely encircled with 3–0 silk to lift the vessels to facilitate later occlusion. The occlusion of both common carotid arteries was induced by applying microaneurysm clips (Surgipro Surgical Micro Vessel Clips, 50–80 g closing pressure) on each vessel for a period of 20minutes followed by the removal of the clips. Sham-operated animals were subjected to the same anesthetic and surgical interventions, with the exception that the carotid arteries remained unclipped. The skin incision was then sutured and the animals were maintained the body temperatures at 37°C until their recovery from the anesthesia. Acetated Ringer's solution (0.5 mL) was administered subcutaneously to all the animals 30 minutes and 24 h after ischemia. All mice were housed in a temperature-controlled facility. Fast blood glucose levels were measured at the following time points: before the operation, 3 days post operation, 7 days post operation and 14 days post operation (S1 Fig). 14 days after the surgery, the mice were subjected to experiments.

### Mitochondrial preparation

Mice brain mitochondria were purified as we previously described [24, 66]. Briefly, brain cortex were dissected and placed in ice-cold isolation buffer [225 mM mannitol, 75mMsucrose, 2mM K_2_PO_4_, 0.1%BSA, 5mM Hepes, 1mMEGTA (pH 7.2)] followed by a homogenization for 10 strokes using a Dounce glass homogenizer. The resultant homogenate was centrifuged at 1,300 g for 5 min to remove cell debris, and the supernatant was layered on a 10mL discontinuous gradient of 15% (vol/vol) Percoll and centrifuged at 16,000 rpm on a Sorval RC5C (Rotor SS34) for 10 minutes. The resultant pellet was collected and subsequently resuspended in mitochondria isolation buffer followed by the treatment of 0.2% Digitonin to break synaptosome. After a centrifugation at 8,000 rpm for 10 minutes, the pellet was collected and resuspended in cold mitochondrial isolation buffer. Mitochondrial protein concentration was determined by Bio-Rad protein concentration kit with BSA as standard. The purity of mitochondria was determined as we previously described [25] by using immunoblotting.

### Mitochondrial respiration assays

Mitochondrial respiration assays were performed following our previously published method [24]. Purified mitochondria were energized by glutamate (5mM) and malate (5mM) and subjected to respiration assays on a Clark electrode. Oxygen consumption was triggered by the addition of ADP (25μM). The mitochondrial respiratory control ratio was defined as the ratio of State III respiration/State IV respiration.

### Immunoblotting Analysis

Samples were prepared in 1x sample loading buffer [50 mM Tris-HCl pH 6.8, 2% SDS, 10% glycerol, 1% β-mercaptoethanol, 12.5 mM EDTA and 0.02% bromophenol blue] and proteins were separated by SDS/PAGE (10% or 12% Bis-Tris gel; Life technology), and then transferred to a PVDF membrane for blotting (BioRad Laboratories). After blocking in TBS buffer (20 mM Tris-HCl, 150 mM sodium chloride) containing 5% (wt/vol) nonfat dry milk for 1 h at room temperature, the membrane was then incubated and gently shaken overnight (at 4°C) with primary antibodies. This was followed by the incubation with the appropriate secondary antibody for 1 h at room temperature. The following antibodies were used in this experiment: MFN2 (#9482, Cell Signaling Technology), OPA1 (#612606, BD Transduction Lab), DLP1(#611112, BD Transduction Lab), pDLP1 (#3455, Cell Signaling Technology), VDAC (#4866, Cell Signaling Technology), β-Amyloid (#8243, Cell Signaling Technology), HSP60(#4870, Cell Signaling Technology), Goat anti-mouse IgG HRP conjugated and goat anti-rabbit IgG HRP conjugated (Life technology). Images were collected on BioRad Chemidoc Imaging System. Image J software (National Institutes of Health) was used for data analysis.

### Immunostaining of 4-HNE

For paraffin-embedded brain section, the slides after deparaffinization and rehydration were subjected to antigen retrieval by boiling in citric acid buffer for 15 minutes. After blocking, the slices were incubated with antibodies against 4-HNE (ab48506, Abcam). After washing in PBS, the slices were probed with anti-rabbit IgG conjugated with Alexa 594 (Life technology). Images were collected on a Nikon confocal microscope and analyzed by using Nikon NIS Advanced Research software. A negative control using primary antibody preabsorbed with 4-hydroxy-2-nonenal-diethylacetal (OXIS Research, Inc.) was used to determine the threshold of positive 4-HNE staining. The images were binarized before analysis and the intensity was measured as previously described [67].

### ELISA assay for brain and mitochondrial Aβ Measurement

Mouse cortex or mitochondrial fractions were incubated in 5 M guanidine HCl and 50 mM Tris HCl (pH 8.0) overnight and then subjected to Aβ concentration detection using human Aβ1–40 and Aβ1–42 ELISA kits (Life technology) following the manufacturer’s instructions [24, 25]. The level of Aβ was presented as ng/mg protein.

### Histological Assessment of Aβ Plaque

Mice after anesthesia (Isofluorane) were subjected to intracardiac perfusion[68] using saline followed by 4% paraformaldehyde. Brain tissues were dissected and equilibrated in 30% sucrose. Sections were cut at 20μm on a cryostat (Thermo scientific). After 3 times washing in PBS for 5 minutes each, free-floating tissue sections were incubated with 0.3% H2O2 in PBS for 10 minutes at room temperature to block endogenous peroxidase. After 3 times washiing in PBS, sections were blocked with blocking buffer (5% goat serum, 0.3% Triton-X in PBS) for 60 minutes at room temperature. Sections were then incubated with polyclonal rabbit anti-Aβ (#8243, Cell Signaling Technology) at a 1:1,000 dilution overnight at 4°C followed by the incubation with a biotinylated secondary goat anti-rabbit antibody (Sigma) in a 1:1000 dilution for 1 hour at room temperature. After washing in PBS, the sections were incubated in HRP-conjugated Extraevidin (Sigma) at 1:1,000 for 1 hr at room temperature followed by washing in PBS, then developed with developed with 3,3′-Diaminobenzidine (#D5905, Sigma-Aldrich). Images were collected on an Olympus microscope. The percentage of occupied brain area and the intensity of the plaque were determined for each sample using the Image J program (NIH). Data from 3 slices per brain sample, spaced every 1 mm were averaged.

### In situ detection of mitochondrial superoxide levels

We performed **I**n situ detection of mitochondrial superoxide levels by using Mitosox Red as we previously described [24]. Briefly, MitoSox Red (Life Technology) at 1mg/ kg body weight was intravenously injected via the mouse tail vein. 30 min after the injection, the mice were anesthetized the mice and sacrificed by transcardial perfusion with cold saline and then cold, freshly prepared 3.7% paraformaldehyde. The brain tissue was quickly dissected and frozen in 2-methyl butane (Sigma) with dry ice. Coronal frozen brain sections were prepared followed by a blocking using 5% BSA. The slices were incubated with antibody against NeuN (Millipore). After washing in PBS, the slices were probed with anti-mouse IgG conjugated with Alexa 488 (Life technology). We then examined sections under a fluorescence microscope immediately after the mounting. The images were binarized for the analysis of MitoSox Red intensity. Brain sections from mice were blindly coded and processed in parallel. Codes were broken after the analysis was complete.

### Morris Water Maze

Morris water maze was conducted to access the mice spatial learning and reference memory according to previously described protocol [69]. Briefly, mice were trained to find a submerged escape platform in an open swimming arena. Repeated trials (n = 4) were performed each day for 9 days by starting the mice at different non-congruent start locations (NW, N, NE, E, SE) while keeping the platform at a single location (SW). Each trial lasted 60 seconds with an additional 30 seconds learning time where mice were allowed to remain on the platform. After 9 days learning, mice were subjected to a probe test in which the platform was removed. Mice were analyzed for number of times they passed previous learning time platform location (SW). Behavior data was analyzed using HVS Image 2015 software (HVS Image).

### Mouse neuron culture and treatment

Neurons were cultured as previously described [25]. Briefly, mouse primary neurons were cultured in neuron culture medium (neurobasal A with 2% B27 supplement, 0.5mM L-glutamine, 50U/ml penicillin, and 50μg/ml streptomycin) with an appropriate density. Neurons at Div 7 days were exposed to vehicle or oligomeric Aβ (200 nM) in the absence of presence of mitotempo (10 μM, Sigma) for a co-incubation of 24 hours followed by glucose- and oxygen-deprivation (OGD) [70]. Neurons were washed with PBS and the culture medium was replaced with glucose-free Earle’s balanced salt solution (EBSS) with vehicle or oligomeric Aβ treatment in the absence of presence of mitotempo. Cultured neurons were then placed in an anoxic chamber (NAPCO, Precision Scientific) filled with 5% CO_2_, 5% H_2_ and 90% N_2_ at 37°C for 1 h. Oxygen levels were maintained below 1% O_2_. Control neurons were washed with PBS and the culture medium was replaced with glucose-containing EBSS with vehicle or oligomeric Aβ treatment in the absence or presence of mitotempo in a regular 5% CO_2_ cell culture incubator. After the treatment, the neurons were then maintained in regular culture medium with or without Mitotempo in a regular 5% CO_2_ incubator.

### TUNEL assay

TUNEL assay was performed by using in situ colorimetric cell death assay kits from Promega following the manufacturer's instruction. The extent of brain damage was represented as number of TUNEL-positive cells per mm^2^ and the cell death of cultured neurons was evaluated by the percentage of TUNEL-positive neurons.

### Oligomeric Aβ preparation

Oligomeric Aβ1–42 was prepared as previously described [71].

### Mitochondrial superoxide assay

Mitochondrial superoxide was determined as previously described by using Mitosox Red (Life technologies)[22–24]. Neurons were incubated with 2 μM MitoSox Red and 200 nM mitotracker green (Life Technology) for 30 minutes followed by washing. The images of Mitosox Red staining were collected on a Nikon inverted confocal microscope with on stage incubator. The intensity was subsequently analyzed by using Nikon NIS Advanced Research software.

### Statistics

Two-way ANOVA followed by Bonferroni post hoc analysis or Student t tests wherever appropriate were used for repeated measure analysis on SPSS software (IBM software). The distribution and variance were normal and similar in all groups. P < 0.05 was considered significant. All data were expressed as the mean ± SEM.

## Supporting Information

S1 Fig Fast blood glucose of sham-operated and ischemic mice. Fast blood glucose levels were measured in the four groups of mice before operation and at 3, 7 and 14 days post-operation by using blood glucose meter (Roche Diagnostics). There was no significant difference in the fast blood glucose levels between each group at any indicated time point.
(TIF)
