# Supplementary figures and images for: Transient Cerebral Ischemia Promotes Brain Mitochondrial Dysfunction and Exacerbates Cognitive Impairments in Young 5xFAD Mice
Source: PLoS One. 2015 Dec 3;10(12):e0144068. doi: 10.1371/journal.pone.0144068 (PMC4669173; doi:10.1371/journal.pone.0144068)

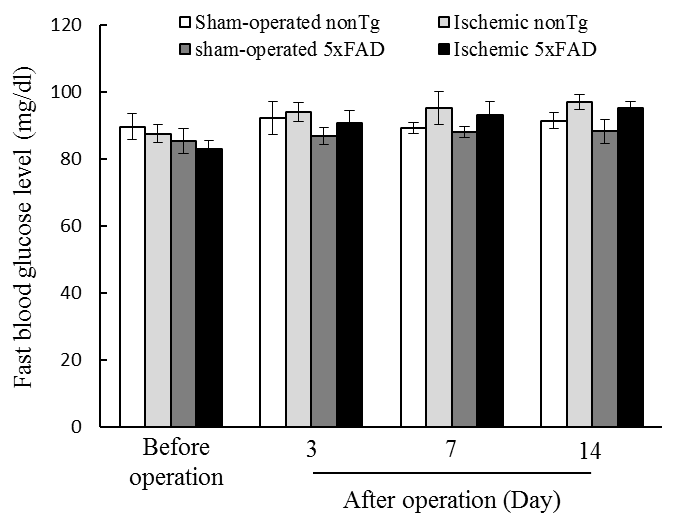

Supplement: S1 Fig — Fast blood glucose levels were measured in the four groups of mice before operation and at 3, 7 and 14 days post-operation by using blood glucose meter (Roche Diagnostics). There was no significant difference in the fast blood glucose levels between each group at any indicated time point. (TIF) [file pone.0144068.s001.tif]
